# Screw and cement augmentation of patella defects in knee arthroplasty

**DOI:** 10.1308/rcsann.2014.96.1.78

**Published:** 2014-01

**Authors:** N Jayasekera, A Lakdawala, AD Toms, KS Eyres

**Affiliations:** Exeter Knee Reconstruction Unit (EKRU), Royal Devon and Exeter NHS Foundation Trust,UK

## BACKGROUND

At primary and revision knee arthroplasty, a deficient patella with a contained or uncontained defect is often encountered,^1,2^ especially in the presence of longstanding maltracking. In these cases, reconstruction is difficult, leaving an unsupported implant. Following the good outcomes achieved using cement fixation of large defects of the tibial plateau augmented with screws,^3^ we describe a technique to address patella defects in cases where patella tracking is a problem and would compromise the final result.

## TECHNIQUE

**Figure 1:**
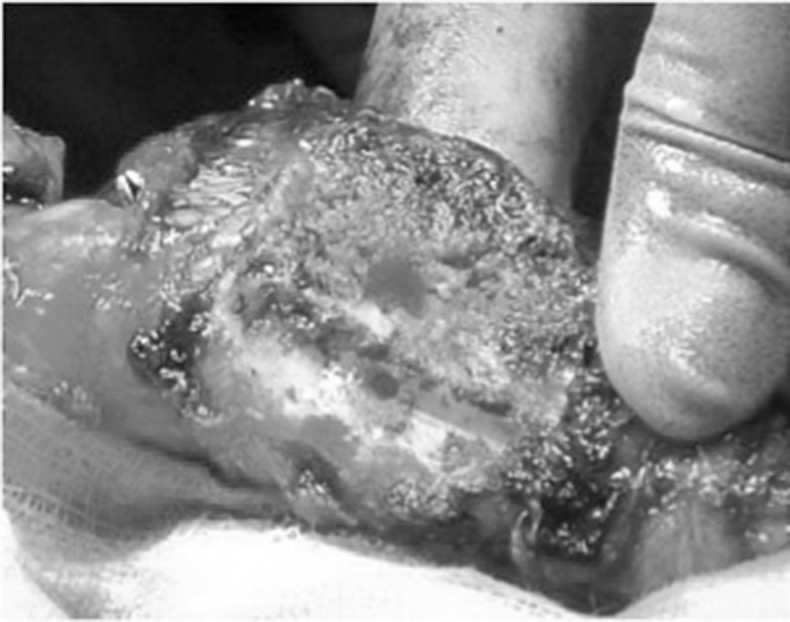
Clinical photograph of prepared patella with uncontained defect

**Figure 2:**
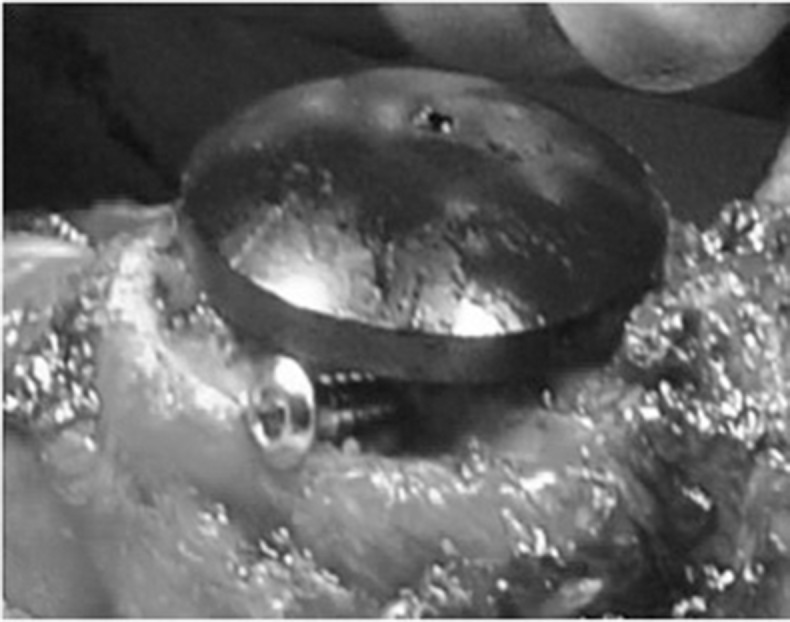
Clinical photograph of screw placement and patella trial component

**Figure 3:**
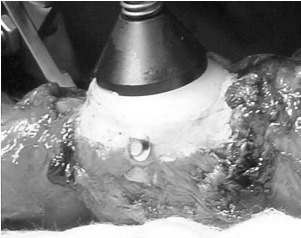
Clinical photograph of cemented patella component with screw augmentation

**Figure 4:**
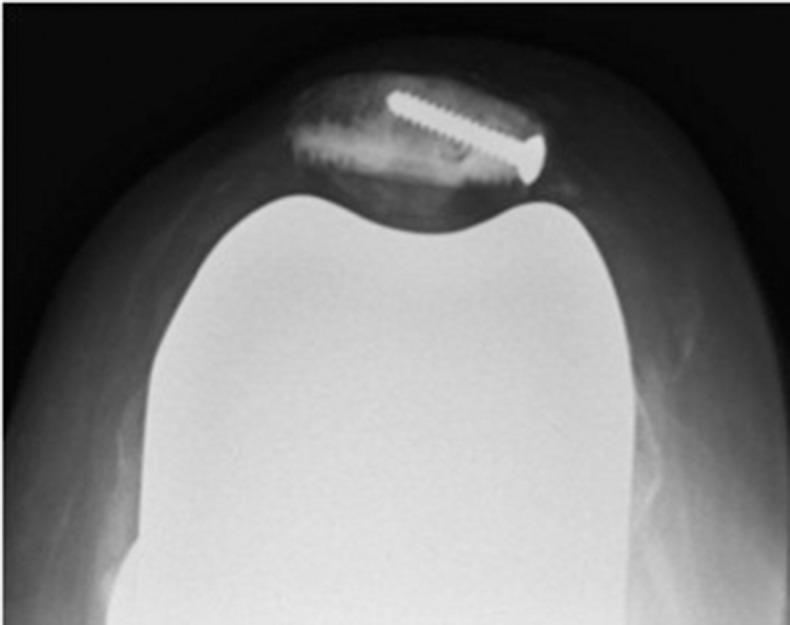
Radiography of the reconstructed patella at five-year follow-up appointment

After conservative bone resection of the patella, the residual defect can be assessed and augmented by screws using a 2.5mm drill to secure a desired number (one to four) of standard 3.5mm cortical screws in the defect. These are orientated to provide maximum support and also to enable placement of the patella trial component (Figs 1 and 2). The patella component is then cemented with the screw used as a scaffold to augment fixation to the bone (Figs 3 and 4).

## DISCUSSION

In cases where the surgeon would prefer to resurface a deficient patella because the option of retaining the host would lead to maltracking, we have found this technique to be reliable, versatile and cheap to overcome the otherwise complicated scenario of patella deficiency. It avoids the risk of fracture by reducing the amount of bone that would otherwise be resected for a traditional fixation, and obviates the need for more cumbersome and expensive augmented patella implants, which have a poor track record.^4,5^

## CONFLICT OF INTERESTS

ADT receives personal research and department support from Stryker, Smith & Nephew and Corin.
